# ﻿Phylogeny of the genus *Loxospora* s.l. (Sarrameanales, Lecanoromycetes, Ascomycota), with *Chicitaea* gen. nov. and five new combinations in *Chicitaea* and *Loxospora*

**DOI:** 10.3897/mycokeys.102.116196

**Published:** 2024-02-19

**Authors:** Łucja Ptach-Styn, Beata Guzow-Krzemińska, James C. Lendemer, Tor Tønsberg, Martin Kukwa

**Affiliations:** 1 Department of Plant Taxonomy and Nature Conservation, Faculty of Biology, University of Gdańsk, Wita Stwosza 59, PL-80-308 Gdańsk, Poland University of Gdańsk Gdańsk Poland; 2 Department of Botany, Research and Collections, CEC 3140, The New York State Museum, 222 Madison Ave., Albany NY 12230, USA The New York State Museum Albany United States of America; 3 Department of Natural History, University Museum, University of Bergen, Allegt. 41, 7800, 5020 Bergen, Norway University of Bergen Bergen Norway

**Keywords:** Lichenised fungi, mtSSU, nuITS, phylogeny, RPB1, Sarrameanaceae, secondary metabolites, sorediate lichens, sterile lichens, taxonomy

## Abstract

*Loxospora* is a genus of crustose lichens containing 13 accepted species that can be separated into two groups, based on differences in secondary chemistry that correlate with differences in characters of the sexual reproductive structures (asci and ascospores). Molecular phylogenetic analyses recovered these groups as monophyletic and support their recognition as distinct genera that differ in phenotypic characters. Species containing 2’-*O*-methylperlatolic acid are transferred to the new genus, *Chicitaea* Guzow-Krzem., Kukwa & Lendemer and four new combinations are proposed: *C.assateaguensis* (Lendemer) Guzow-Krzem., Kukwa & Lendemer, *C.confusa* (Lendemer) Guzow-Krzem., Kukwa & Lendemer, *C.cristinae* (Guzow-Krzem., Łubek, Kubiak & Kukwa) Guzow-Krzem., Kukwa & Lendemer and *C.lecanoriformis* (Lumbsch, A.W. Archer & Elix) Guzow-Krzem., Kukwa & Lendemer. The remaining species produce thamnolic acid and represent *Loxospora* s.str. Haplotype analyses recovered sequences of *L.elatina* in two distinct groups, one corresponding to *L.elatina* s.str. and one to *Pertusariachloropolia*, the latter being resurrected from synonymy of *L.elatina* and, thus, requiring the combination, *L.chloropolia* (Erichsen) Ptach-Styn, Guzow-Krzem., Tønsberg & Kukwa. Sequences of *L.ochrophaea* were found to be intermixed within the otherwise monophyletic *L.elatina* s.str. These two taxa, which differ in contrasting reproductive mode and overall geographic distributions, are maintained as distinct, pending further studies with additional molecular loci. Lectotypes are selected for *Lecanoraelatina*, *Pertusariachloropolia* and P.chloropoliaf.cana. The latter is a synonym of *Loxosporachloropolia*. New primers for the amplification of mtSSU are also presented.

## ﻿Introduction

Lichens are specialised fungi that associate in symbiotic relationships with photoautotrophic partners, termed photobionts, which are mainly represented by green microalgae or cyanobacteria (Büdel and Scheidegger 2008). Numerous lichenised fungi have developed special vegetative diaspores (usually isidia and soredia), which allow the co-dispersal of symbiotic partners and maintenance of the symbiosis (Poelt 1970; Werth and Scheidegger 2012; Sanders 2014; Onuț-Brännström et al. 2018). Lichen species that produce specialised vegetative diaspores are frequently sterile, rarely producing sexual reproductive structures and ascospores (Poelt 1970). This complicates, especially in the case of taxa with crustose thalli, the determination of their systematic position and can render identification difficult due to the scarcity of diagnostic morphological characters (e.g. Ekman and Tønsberg (2002); Kukwa and Pérez-Ortega (2010); Hodkinson and Lendemer (2012, 2013); Guzow-Krzemińska et al. (2017, 2018, 2019); Malíček et al. (2018); Orange (2020); Kukwa et al. (2023)).

Some species that produce lichenised vegetative diaspores are morphologically (except for the development of such diaspores) and chemically almost identical to the taxa that lack those structures and such cases are referred to as species pairs (Poelt 1970; Crespo and Pérez-Ortega 2009). Molecular phylogenetic studies of such pairs and of species with lichenised vegetative diaspores generally, however, suggest that the situation is more complex and nuanced than binary pairs of species that either lack vegetative diaspores and are sexually reproducing or produce vegetative diaspores and are only infrequently sexually reproducing. In some cases, neither species delimited by the presence or absence of vegetative diaspores was found to be monophyletic and, instead, representatives of each were intermingled suggesting that independent lineages do not correspond to reproductive mode (e.g. Lohtander et al. (1998); Buschbom and Mueller (2006); Myllys et al. (2011); Tehler et al. (2013); Ertz et al. (2018)). In other cases, such pairs of species have been recovered as reciprocally monophyletic and sister (e.g. Miadlikowska et al. (2011); Lendemer and Harris (2014); Yakovchenko et al. (2017); Ohmura (2020)). Further, there are recent examples where next generation sequence data have provided support for species pair delimitations that lacked support from analyses of traditionally used loci that are typically more conserved and fewer in number (e.g. Grewe et al. (2018)).

The genus *Loxospora* A. Massal. was described by Massalongo (1852) and, at present, includes thirteen accepted species (Kalb and Hafellner 1992; Kantvilas 2000; Lumbsch et al. 2007; Lendemer 2013; Lücking et al. 2017; Guzow-Krzemińska et al. 2018). *Loxospora* species have been reported from many regions globally (e.g. Kalb and Hafellner (1992); Kantvilas (2000); Lumbsch et al. (2007); Papong et al. (2009); Kelly et al. (2011); Lendemer (2013); Hafellner and Türk (2016); Berger et al. (2018); Guzow-Krzemińska et al. (2018); Wirth et al. (2018); Marthinsen et al. (2019); Urbanavichus et al. (2020); Westberg et al. (2021)). The genus is classified at present in Sarrameanales B.P. Hodk. & Lendemer in Lecanoromycetes O.E. Erikss. & Winka (Lücking et al. 2017). Previous molecular phylogenetic studies have recovered *Loxospora* to form a well-supported clade, with members divided into two distinct clades (Lumbsch et al. 2007; Lendemer 2013; Guzow-Krzemińska et al. 2018). The species in one clade are characterised by asci having uniformly amyloid apical dome, septate, fusiform to ellipsoidal ascospores and the production of thamnolic acid as the main secondary metabolite (Hafellner 1984; Kantvilas 2000; Guzow-Krzemińska et al. 2018). This clade corresponds to *Loxospora* s.str. and contains the type species, *L.elatina* (Ach.) A. Massal. (Massalongo 1852; Galloway 2007). The second clade comprises four species producing 2’-*O*-methylperlatolic acid (Lumbsch et al. 2007; Lendemer 2013; Guzow-Krzemińska et al. 2018). Ascomata are known only in one of those species, *L.lecanoriformis* Lumbsch, A.W. Archer & Elix and, in that taxon, the asci lack an amyloid apical dome and have simple ascospores (Lumbsch et al. 2007; Papong et al. 2009). The chemical and anatomical characters, especially the ascus apical dome amyloidy, combined with the monophyletic resolution as distinct from *Loxospora* s.str., suggest that this latter group merits recognition at the genus level.

In summer 2021, while performing field lichen studies in northern Poland, we collected specimens resembling *Loxosporaelatina* growing on bark of *Alnusglutinosa* in black alder forest. They contained thamnolic acid as the main secondary metabolite; however, the thallus was continuous to areolate, in contrast to the tuberculate thalli typically found in *L.elatina* (e.g. Stenroos et al. (2016)). Molecular analyses showed that these specimens and some other samples published by Kelly et al. (2011) formed a group distinct from samples of *L.elatina* with typical tuberculate thalli. Recognising the need to re-evaluate the delimitation of *L.elatina* based on this material, we analysed additional sequences and specimens of other *Loxospora* species to confirm the relationships amongst currently recognised species, especially *L.ochrophaea* (Tuck.) R.C.Harris, which has been presumed to be the strictly sexual, esorediate counterpart to *L.elatina* (Brodo et al. 2001; Guzow-Krzemińska et al. 2018). Based on these analyses, we recognise the material of *L.elatina* with continuous to areolate thalli as distinct and introduce a new combination for it, discuss the status of *L.elatina* s.str. and *L.ochrophaea* (Tuck.) R.C. Harris and introduce the genus *Chicitaea* for the clade of *Loxospora* species producing 2’-*O*-methylperlatolic acid, which necessitates four new combinations.

## ﻿Materials and methods

### ﻿Taxon sampling

Lichen material was studied from BG, BM, BILAS, E, HBG, H-ACH, NY, O, UGDA and herb. Maliček. Morphology was examined using a Nikon SMZ 800N stereomicroscope. Secondary lichen metabolites were studied by thin layer chromatography (TLC) (Culberson and Kristinsson 1970; Orange et al. 2001). For reference of squamatic acid and thamnolic acid, we used extracts from *Cladoniaglauca* Flörke and *C.digitata* (L.) Baumg., respectively.

### ﻿DNA extraction, PCR amplification and DNA sequencing

Small pieces of thalli (approx. 2 mm^2^) were put into Eppendorf tubes. Then DNA was extracted using a GeneMATRIX Plant & Fungi DNA Purification Kit (EURX) or a modified CTAB method (Guzow-Krzemińska and Węgrzyn 2000). Sequences of three molecular markers were amplified: nuITS rDNA using ITS1F (Gardes and Bruns 1993) or ITS5 (White et al. 1990) and ITS4 (White et al. 1990) primers, RPB1 using g-RPB1-A for (Stiller and Hall 1997) and f-RPB1-C rev (Matheny et al. 2002) primers and mtSSU using mrSSU1 (Zoller et al. 1999) and mrSSU3R (Zoller et al. 1999) primers. Due to difficulties in mtSSU amplification, new primers were designed by one of the authors (Beata Guzow-Krzemińska; primers here referred to as “Lox_mtSSU620_For”: 5’-TTTACCTATATGTCTTGACCAA-3’ and “Lox_mtSSU620_Rev”: 5’-CTCTTATCATATTCCAATATAATG-3’). PCR settings for each set of primers are shown in Suppl. material 1. Electrophoresis was performed on a 1% agarose gel to determine whether amplification of target molecular markers was successful. PCR products were purified using Clean-Up Concentrator (A&A Biotechnology). Sequencing was performed by Macrogen (The Netherlands). All newly-generated sequences were deposited in GenBank and their GenBank Acc. Numbers are presented in Table 1.

**Table 1.** Specimen data and the GenBank accession numbers of newly-obtained sequences of the taxa used in the phylogenetic analyses. A dash provides information about lack of DNA sequence. For sequences obtained from GenBank, see Suppl. material 2.

Species	Origin	Collection and herbarium	GenBank accession numbers		
			nuITS	mtSSU	RPB1
***Chicitaeaconfusa* 3**	U.S.A. North Carolina. Carteret Co.	Lendemer 35738 (NY-1885635)	PP080079	PP080125	–
***Chicitaeaconfusa* 4**	U.S.A. North Carolina. Jones Co.	Lendemer 35691 (NY-1885682)	PP080080	PP080126	–
***Chicitaeaconfusa* 5**	U.S.A. North Carolina. Carteret Co.	Lendemer 35485 (NY-1885425)	PP080081	PP080127	–
**Chicitaeaaff.confusa 6**	U.S.A. North Carolina. Jones Co.	Lendemer 35655 (NY-1885717)	PP080082	PP080128	–
***Chicitaeaconfusa* 7**	U.S.A. North Carolina. Craven Co.	Lendemer 35418 (NY-1885382)	PP080083	PP080129	–
***Chicitaeaconfusa* 8**	U.S.A. North Carolina. Dare Co.	Lendemer 36747 (NY-1885847)	PP080084	–	–
***Chicitaeaconfusa* 9**	U.S.A. North Carolina. Tyrrell Co.	Lendemer 36584 (NY-1886010)	PP080085	–	–
***Chicitaeaconfusa* 10**	U.S.A. North Carolina. Washington Co.	Lendemer 36398 (NY-1886197)	PP080086	–	–
***Chicitaeacristinae* 10**	Poland. Carpathians, Bieszczady	Szymczyk s.n. (UGDA L-60232)	PP080087	PP080130	–
***Loxosporachloropolia* 5**	Poland. Wybrzeże Słowińskie	Ptach-Styn, Kukwa Lox. 1 (UGDA L-60093)	PP080088	–	PP083715
***Loxosporachloropolia* 6**	Poland. Wybrzeże Słowińskie	Ptach-Styn, Kukwa Lox. 2 (UGDA L-60094)	PP080089	–	PP083716
***Loxosporachloropolia* 7**	Poland. Wybrzeże Słowińskie	Ptach-Styn, Kukwa Lox. 3 (UGDA L-60095)	PP080090	–	PP083717
***Loxosporachloropolia* 8**	Poland. Wybrzeże Słowińskie	Ptach-Styn, Kukwa Lox. 4 (UGDA L-60096)	PP080091	–	PP083718
***Loxosporachloropolia* 9**	Poland. Wybrzeże Słowińskie	Ptach-Styn, Kukwa Lox. 5 (UGDA L-60097)	PP080092	–	–
***Loxosporachloropolia* 10**	Poland. Wybrzeże Słowińskie	Ptach-Styn, Kukwa Lox. 6 (UGDA L-60098)	PP080093	–	PP083720
***Loxosporachloropolia* 11**	Poland. Wybrzeże Słowińskie	Ptach et al. B1 (UGDA L-47764)	PP080094	PP080131	PP083721
***Loxosporachloropolia* 12**	Poland. Wybrzeże Słowińskie	Ptach et al. B2 (UGDA L-47765)	PP080095	PP080132	PP083714
***Loxosporachloropolia* 13**	Poland. Wybrzeże Słowińskie	Ptach et al. B3 (UGDA L-47766)	PP080096	PP080133	–
***Loxosporacismonica* 2**	U.S.A. Tennessee. Blount Co.	Lendemer 44526 (NY-2438341)	PP080097	–	–
***Loxosporacismonica* 3**	Canada. New Brunswick. Charlotte Co.	Harris 61785 (NY-2712391)	PP080098	PP080134	–
***Loxosporacismonica* 4**	Romania. Carpathians	Malíček 14899, Steinová (herb. Malíček)	–	PP080135	–
***Loxosporaelatina* 6**	Poland. Carpathians, Bieszczady	Szymczyk s.n. (UGDA L-47757)	PP080099	PP080136	–
***Loxosporaelatina* 7**	Poland. Carpathians, Bieszczady	Szymczyk s.n. (UGDA L-47759)	PP080100	PP080137	–
***Loxosporaelatina* 8**	Poland. Carpathians, Bieszczady	Szymczyk s.n. (UGDA L-47760)	PP080101	PP080138	–
***Loxosporaelatina* 9**	Poland. Carpathians, Bieszczady	Szymczyk s.n. (UGDA L-47761)	PP080102	PP080139	–
***Loxosporaelatina* 10**	Poland. Carpathians, Bieszczady	Szymczyk s.n. (UGDA L-47762)	PP080103	PP080140	–
***Loxosporaelatina* 11**	Poland. Białowieski National Park	Szymczyk 883 (UGDA L-47745)	PP080104	–	–
***Loxosporaelatina* 12**	Poland. Białowieski National Park	Szymczyk 1076 (UGDA L-47746)	PP080105	PP080141	–
***Loxosporaelatina* 13**	Poland. Białowieski National Park	Szymczyk 1085 (UGDA L-47747)	PP080106	–	–
***Loxosporaelatina* 14**	Poland. Białowieski National Park	Szymczyk 1208 (UGDA L-47748)	PP080107	–	–
***Loxosporaelatina* 15**	Poland. Białowieski National Park	Szymczyk 1255 (UGDA L-47750)	PP080108	–	–
***Loxosporaelatina* 16**	Poland. Białowieski National Park	Szymczyk 1295 (UGDA L-47751)	PP080109	PP080142	–
***Loxosporaelatina* 17**	Poland. Równina Bielska	Szymczyk 1405 (UGDA L-47752)	PP080120	–	–
***Loxosporaelatina* 18**	Poland. Równina Bielska	Szymczyk 1464 (UGDA L-47755)	PP080121	–	–
***Loxosporaelatina* 19**	Estonia. Pärnu Co.	Kukwa 20481 (UGDA L-34378)	–	PP080147	–
***Loxosporaelatina* 20**	U.S.A. Maine. Washington Co.	Harris 60661 (NY-1818725)	PP080119	–	–
***Loxosporaelatina* 21**	U.S.A. Michigan Cheboygan Co.	Lendemer 45025 (NY-2439450)	PP080117	–	–
***Loxosporaelatina* 22**	U.S.A. New York. Greene Co.	Lendemer 52960 (NY-3217196)	PP080114	–	–
***Loxosporaelatina* 23**	U.S.A. North Carolina. Haywood Co.	Lendemer 53286 (NY-3218018)	PP080115	–	–
***Loxosporaelatina* 24**	U.S.A. North Carolina. Macon Co.	Lendemer 46493 (NY-2795153)	–	PP080145	–
***Loxosporaelatina* 25**	U.S.A. Tennessee. Sevier Co.	Tripp 5040 (NY-2358356)	PP080110	PP080143	–
***Loxosporaelatina* 26**	Canada. Newfoundland	McCarthy 4138 (NBM)	PP080122	–	PP083719
***Loxosporaelatina* 27**	Canada. Newfoundland	McCarthy 4139 (NBM)	PP080123	–	–
***Loxosporaelatina* 28**	Russia. Caucasus Mts	Malíček et al. 10346 (herb. Malíček)	–	PP080146	–
***Loxosporaelatina* 29**	Czechia. Southern Bohemia	Malíček 14726 (herb. Malíček)	–	PP080148	–
***Loxosporaelatina* 30**	Czechia. Silesia	Malíček et al. 8916 (herb. Malíček)	–	PP080149	–
***Loxosporaelatina* 31**	Russia. Caucasus Mts	Malíček et al. 10515 (herb. Malíček)	–	PP080150	–
***Loxosporaochrophaea* 3**	U.S.A. Maine. Washington Co.	Harris 60662 (NY-1818726)	PP080116	–	–
***Loxosporaochrophaea* 4**	U.S.A. North Carolina. Yancey Co.	Kraus 44 (NY-2607571)	PP080124	–	–
***Loxosporaochrophaea* 5**	U.S.A. North Carolina. Haywood Co.	Lendemer 45473 (NY-2440690)	PP080111	–	–
***Loxosporaochrophaea* 6**	U.S.A. Tennessee. Sevier Co.	Lendemer 47245 (NY-2795450)	PP080112	PP080144	–
***Loxosporaochrophaea* 7**	U.S.A. Tennessee. Sevier Co.	Lendemer 46150 (NY-2606798)	PP080113	PP091207	–
***Loxosporaochrophaea* 8**	U.S.A. Tennessee. Sevier Co.	Lendemer 45684 (NY-2441234)	PP080118	–	–

### ﻿Sequence alignments and phylogenetic analyses

The newly-obtained sequences were trimmed using the Chromas programme (http://technelysium.com.au/wp/). All sequences were analysed using BLASTn search (Altschul et al. 1990). Independent alignments of nuITS, mtSSU rDNA and RPB1 markers were prepared using Seaview software (Galtier et al. 1996; Gouy et al. 2010) employing muscle option and guidance2 software implemented on an online website (Sela et al. 2015; https://guidance.tau.ac.il/). Single locus alignments consisted of 68 nuITS rDNA sequences with 548 sites, 47 mtSSU rDNA sequences with 635 sites and 13 RPB1 sequences with 562 sites. Then, datasets were concatenated into one matrix which consisted of 83 terminals with 1745 positions. The concatenated dataset was subjected to IQ-TREE analysis to find best-fitting nucleotide substitution models for each partition (Nguyen et al. 2015; Chernomor et al. 2016; Kalyaanamoorthy et al. 2017; Hoang et al. 2018). The model selection was restricted to models implemented in MrBayes and the following nucleotide substitution models for the three predefined subsets were selected: HKY+F+I for mtSSU rDNA, K2P+F+G4 for nuITS and K2P+F+I for RPB1. The search for the Maximum Likelihood tree was performed in IQ-TREE and followed with 1000 bootstrap replicates (Nguyen et al. 2015; Chernomor et al. 2016; Kalyaanamoorthy et al. 2017; Hoang et al. 2018).

The Bayesian analysis was conducted using MrBayes 3.2.7a (Huelsenbeck and Ronquist 2001; Ronquist and Huelsenbeck 2003) on the CIPRES Science Gateway (Miller et al. 2010). The analyses were conducted by running 10,000,000 generations. The chain was sampled every 1000^th^ generation. Posterior probabilities (PP) were determined by calculating a majority-rule consensus tree after discarding the initial 25% trees of each chain as the burn-in. All trees were visualised in FigTree v.1.4. (Rambaut 2009) and further modified in Inkscape (https://inkscape.org/). Bootstrap support (BS values ≥ 75) and PP values (values ≥ 0.95) are given near the branches on the phylogenetic tree.

Sequences obtained from GenBank and used in phylogenetic analyses are listed in Suppl. material 2.

### ﻿Preparation of haplotype networks

Moreover, independent alignments of each marker for specimens of *L.elatina*, *L.ochrophaea* and *L.chloropolia* were prepared using Seaview software (Galtier et al. 1996; Gouy et al. 2010) employing muscle option and followed with manual correction. The final nuITS rDNA alignment consisted of 46 sequences with 443 sites, while RPB1 alignment consisted of 11 sequences with 723 sites. Haplotype analyses were performed using PopART software (https://popart.maths.otago.ac.nz) employing TCS network option (Clement et al. 2002). Moreover, variable sites that distinguish these taxa were identified. Similar analyses were done for specimens of *L.assateaguensis*, *L.confusa* and *L.lecanoriformis*. The final alignment of nuITS rDNA consisted of 11 sequences with 534 sites, while mtSSU rDNA alignment consisted of eight sequences and 613 sites.

## ﻿Results and discussion

**Figure 1.**
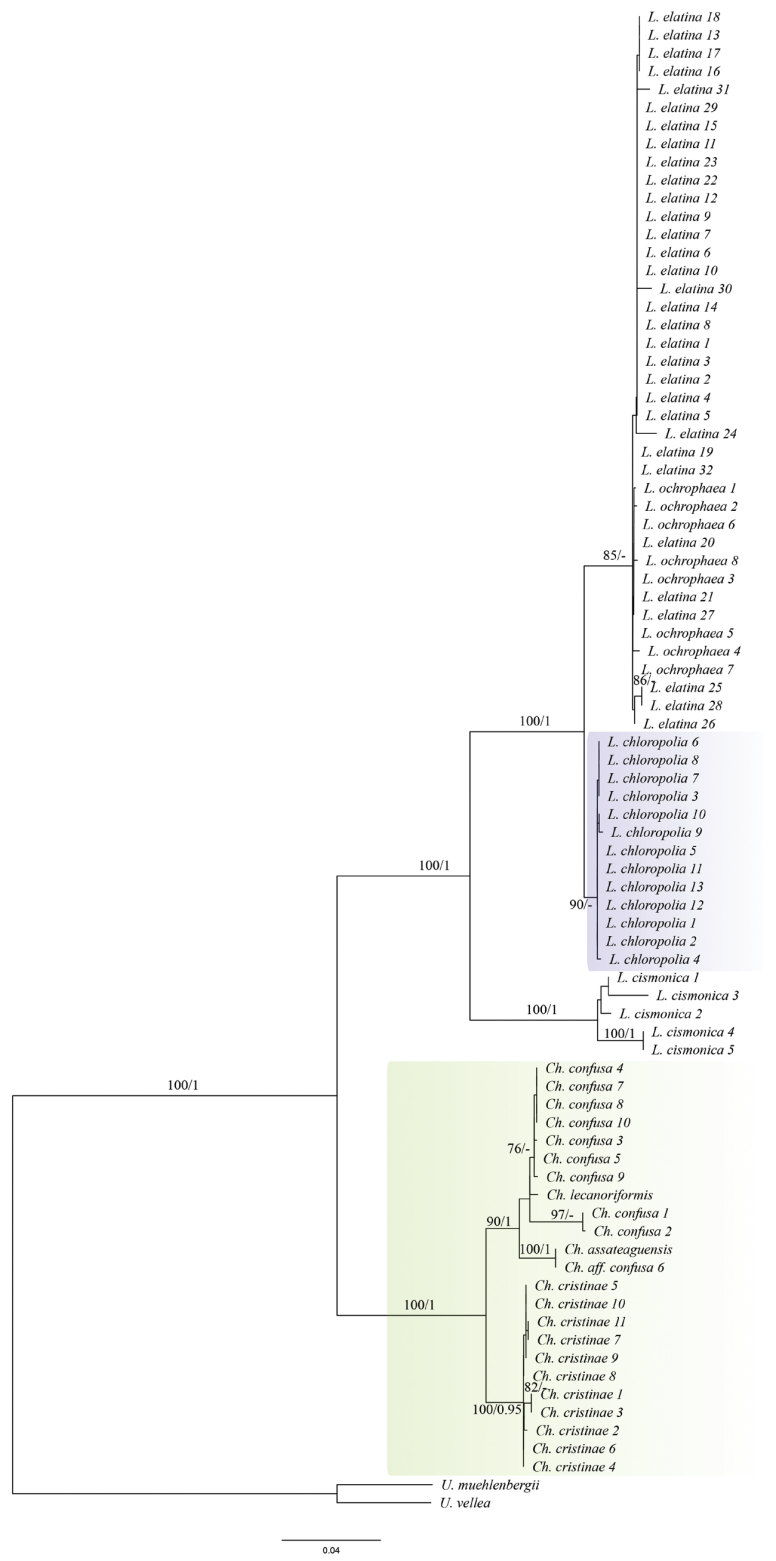
IQ-tree based on a combined nuITS rDNA, mtSSU and RPB1 dataset for *Loxospora* s.l. The names of species are followed with sample number (see Table 1, Suppl. material 2). Bootstrap supports from IQ-tree analysis ≥ 70 (first value) and posterior probabilities from BA ≥ 0.95 (second value) are indicated near the branches. *Umbilicaria* spp. were used as outgroup. *Loxosporachloropolia* clade is marked with blue box and *Chicitaea* gen. nov. is marked with green box.

**Figure 2.**
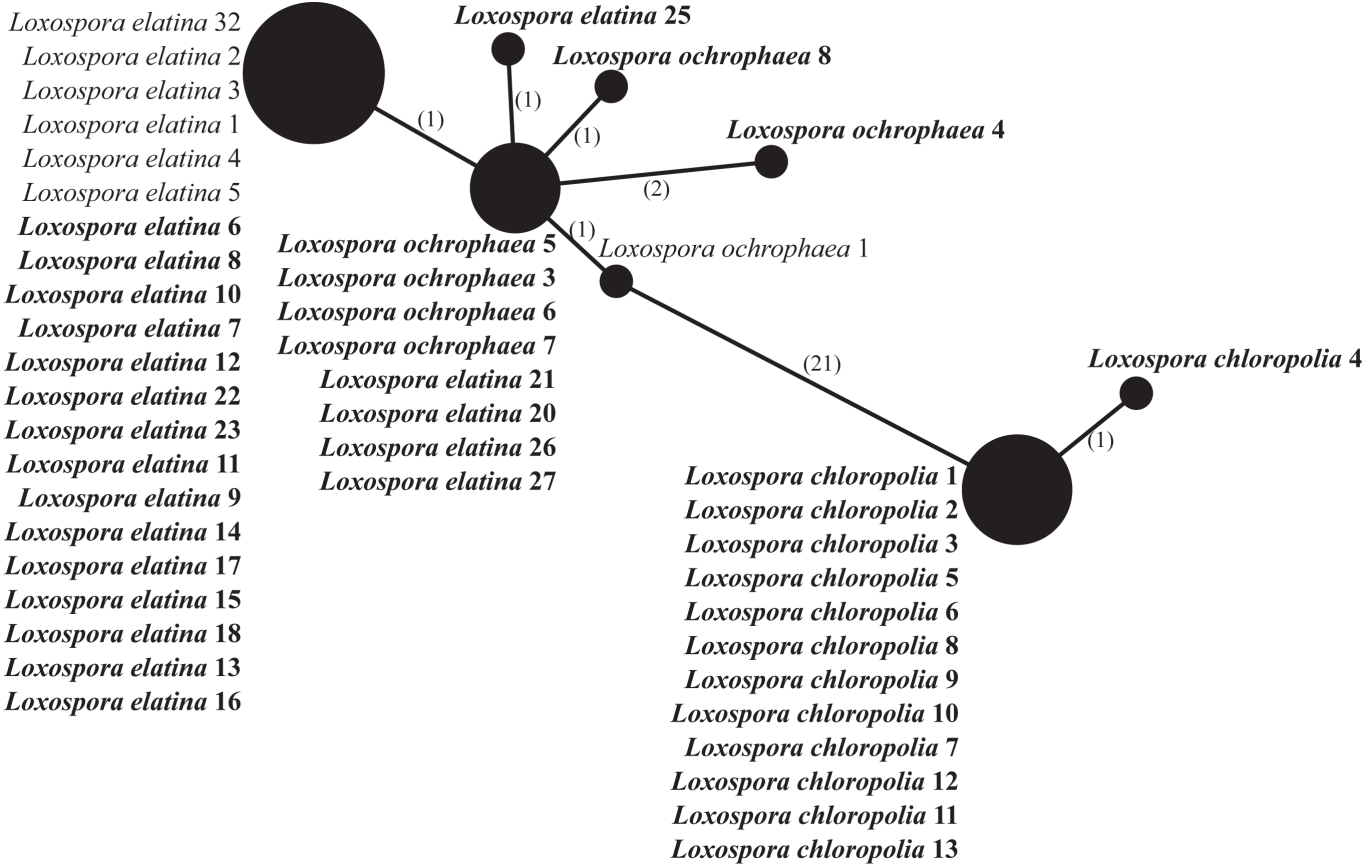
Haplotype network showing relationships between nuITS rDNA sequences from *Loxosporachloropolia*, *L.elatina* and *L.ochrophaea*. The names of species are followed with sample numbers (see Table 1, Suppl. material 2). Newly-sequenced samples are marked in bold. Mutational changes are presented as numbers in brackets near lines between haplotypes.

**Figure 3.**
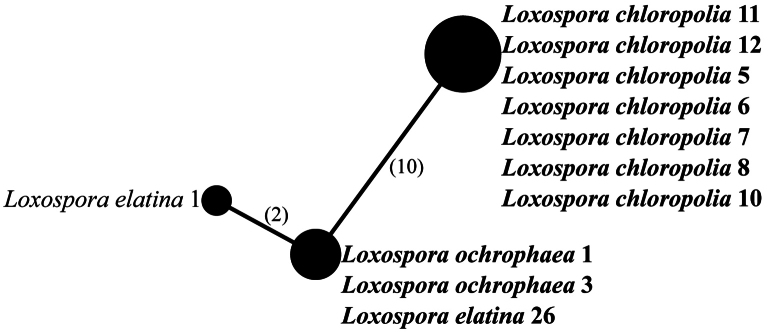
Haplotype network showing relationships between RPB1 sequences from *Loxosporachloropolia*, *L.elatina* and *L.ochrophaea*. The names of species are followed with sample numbers (see Table 1, Suppl. material 2). Newly-sequenced samples are marked in bold. Mutational changes are presented as numbers in brackets near lines between haplotypes.

The representatives of the genus *Loxospora* s.l. are split into two highly-supported major clades (Fig. 1). The larger clade corresponds to *Loxospora* s.str. (type: *L.elatina*), all containing thamnolic acid as the main secondary lichen substance and having asci with a uniformly amyloid apical dome and ascospores that are septate, fusiform to ellipsoidal and somewhat curved or twisted (Tønsberg 1992; Brodo et al. 2001; Sanderson et al. 2008). This clade is divided into two subclades. The smaller one consists of representatives of *L.cismonica* (Beltr.) Hafellner, while the larger subclade consists of two poorly-supported lineages, which might be the result of uneven coverage of sequences for each species in this subclade (see Table 1, Suppl. material 2). However, the phylogenetic analyses, based only on nuITS (not shown here) and the nuITS haplotype network analysis (Fig. 2), recovered these two groups as different and with high confidence. In the nuITS rDNA haplotype network analysis, these groups differ from each other in 21 nucleotide positions and the variability within the groups is up to three substitutions. Moreover, RPB1 haplotype network analysis also supports distinction of these two groups as they differ in 10 positions (Fig. 3), while the mtSSU rDNA marker showed very low variation (data not shown). The larger group includes sequences of specimens with at least partly tuberculate thalli with soralia, which are often fusing (i.e. corresponding to *L.elatina* s.str.) and thalli that uniformly lack soralia, but are typically fertile (i.e. corresponding to *L.ochrophaea*). The smaller group consists of sequences of samples in which the thalli are continuous to slightly cracked-areolate, but never tuberculate and soralia are usually discrete, rarely fusing and, if so, then only in older parts of the thallus.

The specimens whose sequences were recovered in this latter group correspond morphologically to the type material of *Pertusariachloropolia* Erichsen (≡ *Lecanorachloropolia* (Erichsen) Almb.), not to the type of *Lecanoraelatina* Ach. (basionym of *Loxosporaelatina*). *Pertusariachloropolia* was synonymised with *Loxosporaelatina* by Laundon (1963), a treatment followed subsequently by Hafellner and Türk (2016) and Westberg et al. (2021). All of the existing herbarium specimens corresponding to the type of *Pertusariachloropolia* and presented in this present paper were initially identified as *L.elatina* and filed under that name in herbaria. However, as the molecular data show, this material corresponds to a phenotypically distinct monophyletic group for which the name *P.chloropolia* is available. The name is resurrected from synonymy and a new combination is proposed below. The revised circumscriptions of both *Loxosporachloropolia* and *L.elatina* are presented below and lectotypes are selected for both names. Moreover, in addition to morphology, their nuITS rDNA and RPB1 sequences differ in numerous positions of which several may be used as diagnostic characters to distinguish these taxa (Tables 2, 3).

**Table 2.** Variable positions in the alignment of nuITS rDNA marker of *Loxosporachloropolia*, *L.elatina* and *L.ochrophaea*. Variable characters are marked in bold, while diagnostic nucleotide position characters to distinguish *L.chloropolia* from both *L.elatina* and *L.ochrophaea* are marked with a gray background, including indels.

Sequence	Position in alignment																																										
	11	15	16	17	19	20	27	34	35	37	38	42	43	44	45	46	52	68	79	107	311	321	332	334	340	341	342	347	371	387	389	393	401	405	412	416	417	419	420	432	435	439	441
*L.chloropolia* 1	**C**	C	C	**T**	**T**	**T**	**C**	**T**	**A**	**T**	**A**	–	–	–	–	T	**C**	**C**	G	A	**T**	**C**	**G**	**G**	**C**	**T**	**G**	G	**T**	**G**	**T**	**A**	T	**T**	**T**	**T**	T	G	T	–	G	G	G
*L.chloropolia* 2	**C**	C	C	**T**	**T**	**T**	**C**	**T**	**A**	**T**	**A**	–	–	–	–	T	**C**	**C**	G	A	**T**	**C**	**G**	**G**	**C**	**T**	**G**	G	**T**	**G**	**T**	**A**	T	**T**	**T**	**T**	T	G	T	–	G	G	G
*L.chloropolia* 3	**C**	C	C	**T**	**T**	**T**	**C**	**T**	**A**	**T**	**A**	–	–	–	–	T	**C**	**C**	G	A	**T**	**C**	**G**	**G**	**C**	**T**	**G**	G	**T**	**G**	**T**	**A**	**C**	**T**	**T**	**T**	T	G	T	–	G	G	G
*L.chloropolia* 5	**C**	C	C	**T**	**T**	**T**	**C**	**T**	**A**	**T**	**A**	–	–	–	–	T	**C**	**C**	G	A	**T**	**C**	**G**	**G**	**C**	**T**	**G**	G	**T**	**G**	**T**	**A**	Y	**T**	**T**	**T**	T	G	T	–	G	G	G
*L.chloropolia* 6	**C**	C	C	**T**	**T**	**T**	**C**	**T**	**A**	**T**	**A**	–	–	–	–	T	**C**	**C**	G	A	**T**	**C**	**G**	**G**	**C**	**T**	**G**	G	**T**	**G**	**T**	**A**	**C**	**T**	**T**	**T**	T	G	T	–	G	G	G
*L.chloropolia* 8	**C**	C	C	**T**	**T**	**T**	**C**	**T**	**A**	**T**	**A**	–	–	–	–	T	**C**	**C**	G	A	**T**	**C**	**G**	**G**	**C**	**T**	**G**	G	**T**	**G**	**T**	**A**	**C**	**T**	**T**	**T**	T	G	T	–	G	G	G
*L.chloropolia* 9	**C**	C	C	**T**	**T**	**T**	**C**	**T**	**A**	**T**	**A**	–	–	–	–	T	**C**	**C**	G	A	**T**	**C**	**G**	**G**	**C**	**T**	**G**	G	**T**	**G**	**T**	**A**	**C**	**T**	**T**	**T**	T	G	T	–	G	G	G
*L.chloropolia* 10	**C**	C	C	**T**	**T**	**T**	**C**	**T**	**A**	**T**	**A**	–	–	–	–	T	**C**	**C**	G	A	**T**	**C**	**G**	**G**	**C**	**T**	**G**	G	**T**	**G**	**T**	**A**	T	**T**	**T**	**T**	T	G	T	–	G	G	G
*L.chloropolia* 7	**C**	C	C	**T**	**T**	**T**	**C**	**T**	**A**	**T**	**A**	–	–	–	–	T	**C**	**C**	G	A	**T**	**C**	**G**	**G**	**C**	**T**	**G**	G	**T**	**G**	**T**	**A**	**C**	**T**	**T**	**T**	T	G	T	–	G	G	G
*L.chloropolia* 4	**C**	C	C	**T**	**T**	**T**	**C**	**T**	**A**	**T**	**A**	–	–	–	–	T	**C**	**C**	G	A	**T**	**C**	**G**	**G**	**C**	**T**	**G**	G	**T**	**G**	**T**	**A**	T	**T**	**T**	**T**	T	G	T	–	G	**A**	G
*L.chloropolia* 12	**C**	C	C	**T**	**T**	**T**	**C**	**T**	**A**	**T**	–	–	–	–	–	T	**C**	**C**	G	A	**T**	**C**	**G**	**G**	**C**	**T**	**G**	G	**T**	**G**	**T**	**A**	T	**T**	**T**	**T**	T	G	T	–	G	G	G
*L.chloropolia* 11	**C**	C	C	**T**	**T**	**T**	**C**	**T**	**A**	**T**	–	–	–	–	–	T	**C**	**C**	G	A	**T**	**C**	**G**	**G**	**C**	**T**	**G**	G	**T**	**G**	**T**	**A**	T	**T**	**T**	**T**	T	G	T	–	G	G	G
*L.chloropolia* 13	**C**	C	C	**T**	**T**	**T**	**C**	**T**	**A**	**T**	–	–	–	–	–	T	**C**	**C**	G	A	**T**	**C**	**G**	**G**	**C**	**T**	**G**	G	**T**	**G**	**T**	**A**	T	**T**	**T**	**T**	T	G	T	–	G	G	G
*L.ochrophaea* 1	–	C	C	G	A	A	T	C	G	C	**A**	T	C	T	T	T	T	T	G	A	C	T	A	A	T	C	A	G	C	C	**T**	G	T	C	**T**	C	T	G	T	–	G	G	G
*L.ochrophaea* 5	–	C	C	G	A	A	T	C	G	C	**A**	T	C	T	T	T	T	T	G	A	C	T	A	A	T	C	A	G	C	C	C	G	T	C	**T**	C	T	G	T	–	–	G	G
*L.ochrophaea* 3	–	C	C	G	A	A	T	C	G	C	–	T	C	T	T	T	T	T	G	A	C	T	A	A	T	C	A	G	C	C	C	G	T	C	**T**	C	T	G	T	–	G	G	G
*L.ochrophaea* 6	–	C	Y	G	A	A	T	C	G	C	**A**	T	C	T	T	T	T	T	G	A	C	T	A	A	T	C	A	G	C	C	C	G	T	C	**T**	C	T	G	T	–	G	G	G
*L.ochrophaea* 7	–	Y	C	G	A	A	T	C	G	C	**A**	T	C	T	T	T	T	T	G	A	C	T	A	A	T	C	A	G	C	C	C	G	T	C	**T**	C	T	G	T	–	G	G	G
*L.ochrophaea* 8	–	C	C	G	A	A	T	C	G	C	**A**	T	C	T	T	**C**	T	T	G	A	C	T	A	A	T	C	A	G	C	C	C	G	T	C	**T**	C	T	G	T	–	G	G	G
*L.ochrophaea* 4	–	C	C	G	A	A	T	C	G	C	**A**	T	C	T	T	T	T	T	G	**G**	C	T	A	A	T	C	A	G	C	C	C	G	T	C	**T**	C	**C**	G	T	**C**	G	G	G
*L.elatina* 2	–	C	C	G	A	A	T	C	G	C	**A**	T	C	T	T	T	T	T	G	A	C	T	A	A	T	C	A	G	C	C	C	G	T	C	A	C	T	G	T	–	G	G	G
*L.elatina* 3	–	C	C	G	A	A	T	C	G	C	**A**	T	C	T	T	T	T	T	G	A	C	T	A	A	T	C	A	G	C	C	C	G	T	C	A	C	T	G	T	–	G	G	G
*L.elatina* 1	–	C	C	G	A	A	T	C	G	C	**A**	T	C	T	T	T	T	T	G	A	C	T	A	A	T	C	A	G	C	C	C	G	T	C	A	C	T	G	T	–	G	G	G
*L.elatina* 4	–	C	C	G	A	A	T	C	G	C	**A**	T	C	T	T	T	T	T	G	A	C	T	A	A	T	C	A	G	C	C	C	G	T	C	A	C	T	G	T	–	G	G	G
*L.elatina* 5	–	C	C	G	A	A	T	C	G	C	**A**	T	C	T	T	T	T	T	G	A	C	T	A	A	T	C	A	G	C	C	C	G	T	C	A	C	T	G	T	–	G	G	G
*L.elatina* 6	–	C	C	G	A	A	T	C	G	C	–	T	C	T	T	T	T	T	G	A	C	T	A	A	T	C	A	G	C	C	C	G	T	C	A	C	T	G	T	–	G	G	G
*L.elatina* 8	–	C	C	G	A	A	T	C	G	C	–	T	C	T	T	T	T	T	G	A	C	T	A	A	T	C	A	G	C	C	C	G	T	C	A	C	T	G	T	–	G	G	G
*L.elatina* 10	–	C	C	G	A	A	T	C	G	C	–	T	C	T	T	T	T	T	G	A	C	T	A	A	T	C	A	G	C	C	C	G	T	C	A	C	T	G	T	–	G	G	G
*L.elatina* 7	–	C	C	G	A	A	T	C	G	C	–	T	C	T	T	T	T	T	G	A	C	T	A	A	T	C	A	G	C	C	C	G	T	C	A	C	T	G	T	–	G	G	G
*L.elatina* 12	–	C	C	G	A	A	T	C	G	C	–	T	C	T	T	T	T	T	G	A	C	T	A	A	T	C	A	G	C	C	C	G	T	C	A	C	T	G	T	–	G	G	G
*L.elatina* 22	–	C	C	G	A	A	T	C	G	C	–	T	C	T	T	T	T	T	G	A	C	T	A	A	T	C	A	G	C	C	C	G	T	C	A	C	T	G	T	–	G	G	G
*L.elatina* 23	–	C	C	G	A	A	T	C	G	C	–	T	C	T	T	T	T	T	G	A	C	T	A	A	T	C	A	G	C	C	C	G	T	C	A	C	T	G	T	–	G	G	G
*L.elatina* 11	–	C	C	G	A	A	T	C	G	C	–	T	C	T	T	T	T	T	G	A	C	T	A	A	T	C	A	G	C	C	C	G	T	C	A	C	T	G	T	–	G	G	G
*L.elatina* 9	–	C	C	G	A	A	T	C	G	C	–	T	C	T	T	T	T	T	G	A	C	T	A	A	T	C	A	G	C	C	C	G	T	C	A	C	T	G	T	–	G	G	G
*L.elatina* 21	–	C	C	G	A	A	T	C	G	C	**A**	T	C	T	T	T	T	T	G	A	C	T	A	A	T	C	A	G	C	C	C	G	T	C	**T**	C	T	G	T	–	G	G	G
*L.elatina* 20	–	C	C	G	A	A	T	C	G	C	–	T	C	T	T	T	T	T	G	A	C	T	A	A	T	C	A	G	C	C	C	G	T	C	**T**	C	T	G	T	–	G	G	G
*L.elatina* 14	–	C	C	G	A	A	T	C	G	C	–	T	C	T	T	T	T	T	G	A	C	T	A	A	T	C	A	G	C	C	C	G	T	C	A	C	T	G	T	–	G	G	G
*L.elatina* 17	–	C	C	G	A	A	T	C	G	C	–	T	C	T	T	T	T	T	G	A	C	T	A	A	T	C	A	G	C	C	C	G	T	C	A	C	T	G	T	–	G	G	G
*L.elatina* 15	–	C	C	G	A	A	T	C	G	C	–	T	C	T	T	T	T	T	G	A	C	T	A	A	T	C	A	G	C	C	C	G	T	C	A	C	T	G	T	–	G	G	G
*L.elatina* 18	–	C	C	G	A	A	T	C	G	C	–	T	C	T	T	T	T	T	G	A	C	T	A	A	T	C	A	G	C	C	C	G	T	C	A	C	T	G	T	–	G	G	–
*L.elatina* 13	–	C	C	G	A	A	T	C	G	C	–	T	C	T	T	T	T	T	G	A	C	T	A	A	T	C	A	G	C	C	C	G	T	C	A	C	T	G	T	–	G	G	G
*L.elatina* 16	–	C	C	G	A	A	T	C	G	C	–	T	C	T	T	T	T	T	G	A	C	T	A	A	T	C	A	G	C	C	C	G	T	C	A	C	T	G	T	–	G	G	G
*L.elatina* 26	–	C	C	G	A	A	T	C	G	C	**A**	T	C	T	T	T	T	T	G	A	C	T	A	A	T	C	A	G	C	C	C	G	T	C	**T**	C	T	G	T	–	G	G	G
*L.elatina* 27	–	C	C	G	A	A	T	C	G	C	**A**	T	C	T	T	T	T	T	G	A	C	T	A	A	T	C	A	G	C	C	C	G	T	C	**T**	C	T	G	T	–	G	G	G
*L.elatina* 25	–	C	Y	G	A	A	T	C	G	C	**A**	T	C	T	T	T	T	T	S	A	C	T	A	A	T	C	A	K	C	C	C	G	T	C	**T**	C	T	S	Y	–	G	**C**	G
*L.elatina* 32	–	C	C	G	A	A	T	C	G	C	**A**	T	C	T	T	T	T	T	G	A	C	T	A	A	T	C	A	G	C	C	C	G	T	C	A	C	T	G	T	–	G	G	G

**Table 3.** Variable positions in the alignment of RPB1 marker of *Loxosporachloropolia*, *L.elatina* and *L.ochrophaea*. Variable characters are marked in bold, while diagnostic nucleotide position characters to distinguish *L.chloropolia* from both *L.elatina* and *L.ochrophaea* are marked with a gray background.

Sequence	Position in alignment																										
	73	82	106	201	282	291	315	342	344	355	357	414	439	441	472	475	507	513	519	527	540	603	687	700	706	711	723
*L.ochrophaea* 1	G	G	T	T	G	T	T	G	A	G	C	A	G	G	A	A	G	G	A	A	A	T	T	A	C	A	C
*L.ochrophaea* 2	G	G	T	T	G	T	T	G	A	G	C	A	G	G	A	A	G	G	A	A	A	T	T	A	**G**	A	C
*L.elatina* 1	?	?	?	T	G	T	**C**	G	R	G	C	A	G	G	A	A	G	G	A	**G**	A	T	T	N	C	A	C
*L.elatina* 26	G	G	T	T	**K**	T	T	R	A	S	C	A	D	G	R	M	G	G	A	A	A	T	T	A	C	A	C
*L.chloropolia* 11	**A**	**A**	**C**	**C**	G	**C**	T	G	A	G	**T**	**C**	G	**A**	A	A	**A**	**T**	**G**	A	**G**	**C**	**C**	A	C	**G**	**T**
*L.chloropolia* 12	**A**	**A**	**C**	**C**	G	**C**	T	G	A	G	**T**	**C**	G	**A**	A	A	**A**	**T**	**G**	A	**G**	**C**	**C**	A	C	**G**	**T**
*L.chloropolia* 5	**A**	**A**	**C**	**C**	G	**C**	T	G	A	G	**T**	**C**	G	**A**	A	A	**A**	**T**	**G**	A	**G**	**C**	**C**	A	C	**G**	**T**
*L.chloropolia* 6	**A**	**A**	**C**	**C**	G	**C**	T	G	A	G	**T**	**C**	G	**A**	A	A	**A**	**T**	**G**	A	**G**	**C**	**C**	A	C	**G**	**T**
*L.chloropolia* 7	**A**	**A**	**C**	**C**	G	**C**	T	G	A	G	**T**	**C**	G	**A**	A	A	**A**	**T**	**G**	A	**G**	**C**	**C**	A	C	**G**	**T**
*L.chloropolia* 8	**A**	**A**	**C**	**C**	G	**C**	T	G	A	G	**T**	**C**	G	**A**	A	A	**A**	**T**	**G**	A	**G**	**C**	**C**	A	C	**G**	**T**
*L.chloropolia* 10	**A**	**A**	**C**	**C**	G	**C**	T	G	A	G	**T**	**C**	R	**A**	A	A	**A**	**T**	**G**	A	**G**	**C**	?	?	?	?	?

**Figure 4.**
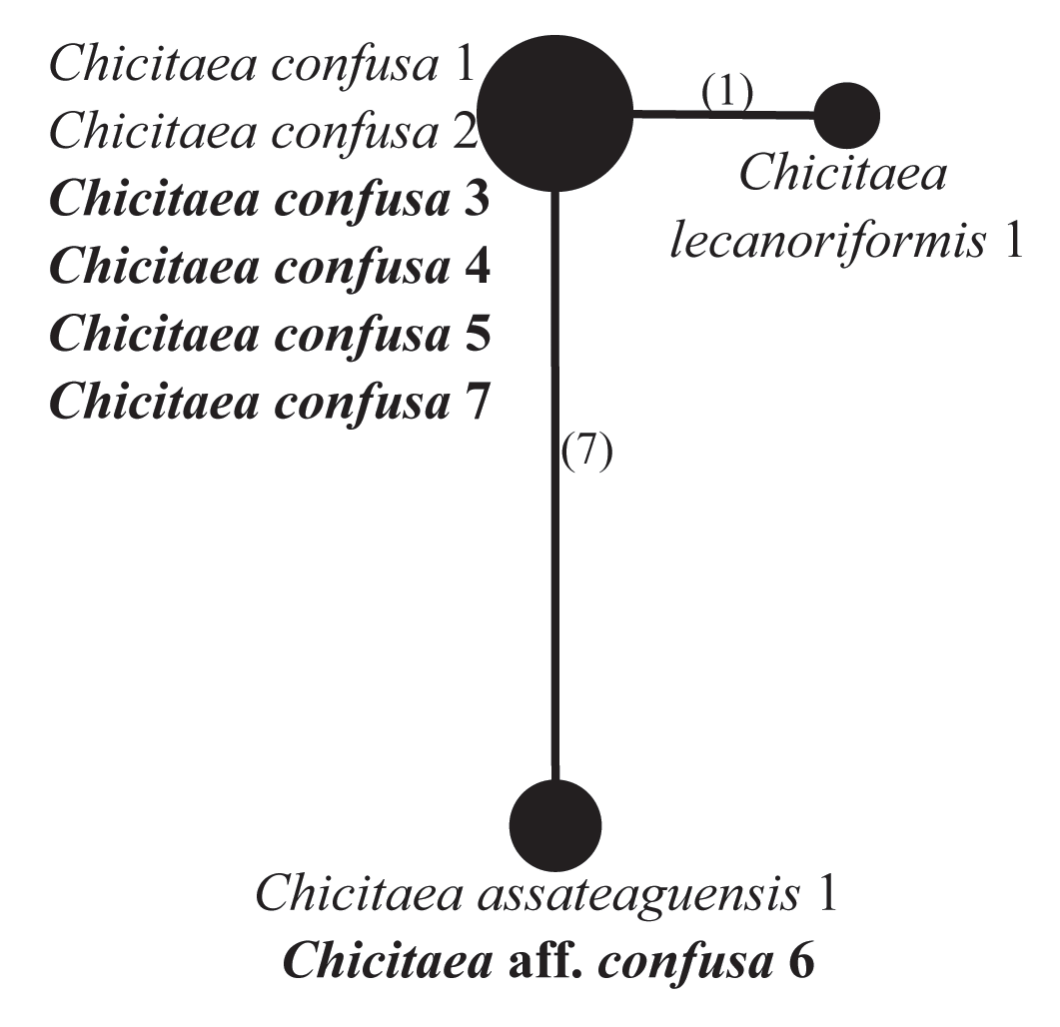
Haplotype network showing relationships between mtSSU rDNA sequences from *Chicitaeaassateaguensis*, *Ch.confusa* and *Ch.lecanoriformis*. The names of species are followed with sample numbers (see Table 1, Suppl. material 2). Newly-sequenced samples are marked in bold. Mutational changes are presented as numbers in brackets near lines between haplotypes.

**Figure 5.**
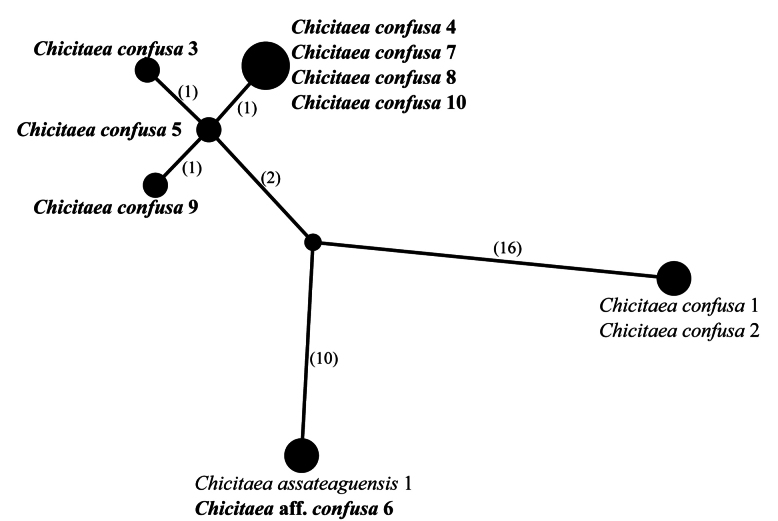
Haplotype network showing relationships between nuITS rDNA sequences from *Chicitaeaassateaguensis* and *Ch.confusa*. The names of species are followed with sample numbers (see Table 1, Suppl. material 2). Newly-sequenced samples are marked in bold. Mutational changes are presented as numbers in brackets near lines between haplotypes.

The smaller clade of *Loxospora* s.l. is represented by *L.assateaguensis* Lendemer, *L.confusa* Lendemer, *L.cristinae* Guzow-Krzem., Łubek, Kubiak & Kukwa and *L.lecanoriformis* (Fig. 1). All these species produce 2’-*O*-methylperlatolic acid and it has been repeatedly suggested that they represent a group distinct from the thamnolic acid producing species of *Loxospora* s. str. which likely merits recognition as a distinct genus (Lumbsch et al. 2007; Lendemer 2013; Guzow-Krzemińska et al. 2018). While apothecia are known only in *L.lecanoriformis*, in that species, the asci lack an amyloid apical dome, unlike in *Loxospora* s.str. and the ascospores are simple, ellipsoidal, straight or slightly bent (Lumbsch et al. 2007; Papong et al. 2009). Due to the consistent differences from *Loxospora* s.str. in secondary lichen substances, the differences in ascus amyloidy and the strongly-supported monophyly of this group in molecular phylogenetic analyses, we recognise it as a distinct genus under the name *Chicitaea* below. Four new combinations are proposed for the species currently known to belong to this clade. *Chicitaeacristinae* was recovered as monophyletic and sister to the rest of the species, which form a well-supported clade, but with poorly resolved relationships between *Ch.confusa* and *Ch.lecanoriformis*. The fertile *Ch.lecanoriformis*, known from Australia and Thailand (Lumbsch et al. 2007; Papong et al. 2009), is nested within a subclade of sequences of *Ch.confusa*, an isidioid species which occurs in North America and is not known to occur in the Southern Hemisphere or Australasia (Lendemer 2013). Due to the lack of nuITS rDNA sequence for *Ch.lecanoriformis* and very low variation found in mtSSU sequences (Fig. 4), the relationship between these species cannot be resolved. Nevertheless, both species clearly differ morphologically and have disjunctive distributions (Lumbsch et al. 2007; Papong et al. 2009; Lendemer 2013). *Chicitaeaconfusa* seems to be paraphyletic and may represent two cryptic species (Fig. 1). This conclusion is also supported by the haplotype analyses of mtSSU and nuITS sequences (Figs 4, 5) which also show that two specimens (*Ch.confusa* 1 and 2) significantly differ from all the newly-sequenced representatives of *Ch.confusa*, but more material is needed to solve this problem. The sequences of one specimen, initially determined as *Ch.confusa* (Ch.aff.confusa 6; Figs 1, 4, 5), is identical in mtSSU and nuITS sequences with *Ch.assateaguensis*. This suggests that *Ch.assateaguensis* can represent a cryptic species, even though, as stated by Lendemer (2013), the species differed from *Ch.confusa*, but more material is necessary before final conclusions.

*Loxosporaelatina* s.str. and *L.ochrophaea* are morphologically similar in terms of thallus and apothecia and both produce thamnolic acid often with elatinic acid and trace amounts of squamatic acid (Tønsberg 1992; Brodo et al. 2001; Sanderson et al. 2008). The only difference between *L.elatina* s.str. and *L.ochrophaea* is the consistent presence of soralia in *L.elatina* (apothecia are very rare) and the absence of soralia in *L.ochrophaea* which is, instead, consistently fertile and routinely produces apothecia (Kalb and Hafellner 1992; Tønsberg 1992; Brodo et al. 2001; Sanderson et al. 2008). From a phenotypic perspective, these two taxa can be considered a species pair (cf. Poelt (1970); Crespo and Pérez-Ortega (2009)).

Although both species are frequently found on the acidic bark of trees and both are distributed in the Northern Hemisphere, their distributions are divergent and not entirely sympatric. *Loxosporaelatina* is widely distributed in boreal and northern temperate areas of the Northern Hemisphere with oceanic climates (e.g. Sanderson et al. (2008); Urbanavichus (2010); Stenroos et al. (2016)). In contrast, *L.ochrophaea* has a narrower, disjunct distribution between the Appalachian-Great Lakes regions of eastern North America and north-eastern Asia (Japan and the Russian Far East) (e.g. Tuckerman (1848); Brodo et al. (2001); Urbanavichus (2010); Ohmura and Kashiwadani (2018)). Indeed, the distributions of these two taxa follow the predictions of the species pair hypothesis, wherein the species with vegetative diaspores has a much larger range compared to that of the strictly sexual species that lacks vegetative diaspores (Poelt 1970; Mattsson and Lumbsch 1989).

In our analyses, sequences of *Loxosporaelatina* s.str. were intermingled with *L.ochrophaea* within the same clade (Fig. 1). Six different nuITS haplotypes were found in these species which differed up to three nucleotide substitutions between each other (Fig. 2). The most common haplotype was found in 20 specimens of *L.elatina* collected in Poland, Switzerland and two geographically distant locations in Appalachian eastern North America (sample *L.elatina* 22 is from New York, U.S.A. and sample *L.elatina* 23 is from North Carolina, U.S.A.; Table 1). Moreover, in the nuITS haplotype network, four samples of *L.elatina* and four samples of *L.ochrophaea* share the same haplotype (Fig. 2). While these samples were all collected in eastern North America, they include samples of each species that were collected at very distant locations (e.g. sample *L.ochrophaea* 3 is from coastal Maine, U.S.A., while samples *L.ochrophaea* 5, 6 and 7 are from Appalachian North Carolina and Tennessee, U.S.A.; sample *L.elatina* 20 is from coastal Maine, U.S.A, sample *L.elatina* 21 is from the Great Lakes of Michigan, U.S.A., while samples *L.elatina* 26 and *L.elatina* 27 are from Newfoundland, Canada; Table 1). Interestingly, a sample of each species was collected in close proximity at the same locality (samples *L.ochrophaea* 3 and *L.elatina* 20, both from the same location on Roque Island in Maine, U.S.A.; Table 1). Given their phenotypic similarity and the lack of resolution using nuITS rDNA, the molecular barcoding marker for fungi, it is possible that *L.elatina* and *L.ochrophaea* may represent variants of a single species. On the other hand, it is also possible that our data were insufficient to distinguish between two closely-related species and more detailed study would allow to find differences between them. Recently, in the case of *Usneaantarctica* Du Rietz and *U.aurantiacoatra* (Jacq.) Bory, RADseq and comparative genomics supported recognition of a species pair that had previously been proposed to be synonyms (Grewe et al. 2018). Given that the species have strongly divergent distributions and that they are morphologically distinct when they co-occur, we refrain from synonymising them at this time.

### ﻿Taxonomy

#### ﻿ Chicitaea

Taxon classificationFungiSarrameanalesSarrameanaceae

Guzow-Krzem., Kukwa & Lendemer gen. nov.

9984B793-BD99-5E20-B5FF-6AF7E5385049

851779

##### Diagnosis.

Differs from *Loxospora* s.str. in the presence of 2’-*O*-methylperlatolic acid (vs. thamnolic acid), asci without an amyloid apical dome (vs. asci with a uniformly amyloid apical dome) and simple, broadly ellipsoid, straight or slightly bent ascospores (known only in the type species; vs. transversely septate ascospores).

##### Generic type.

*Chicitaealecanoriformis* (Lumbsch, A.W. Archer & Elix) Guzow-Krzem., Kukwa & Lendemer.

##### Etymology.

The generic epithet honours Chicita F. Culberson (1931–2023), Senior Research Scientist at Duke University, U.S.A., for her foundational, pioneering and lifelong contributions to the fields of lichen chemistry and lichen taxonomy. In addition to establishing standardised protocols to study lichen secondary chemistry that have been routinely used by workers worldwide for more than half a century, she was an influence for generations of lichenologists with whom she generously shared her knowledge and experience.

##### Description.

Thallus corticolous, pale grey-green to olive-grey, thin or thick, surface smooth to verrucose, sorediate, isidate or without vegetative propagules. Apothecia known in one species, lecanorine, up to 1.5 mm diam., sessile, concave. Thalline margin present, scabrid when young, later entire, dentate, persistent, often flexuose. Disc dark reddish-brown to black, epruinose. Hymenium colourless, inspersed with infrequent oil droplets. Paraphyses simple, unbranched. Hypothecium colourless or pale yellow-brown. Asci claviform to obovate, I–, KI+ slightly blue-green, damaged asci amyloid. Ascospores 6–8 per ascus, broadly ellipsoid, straight or slightly bent, with a single thin wall. Pycnidia found in one species, immersed, visible as minute black dots. Conidia bacilliform.

##### Chemistry.

2’-*O*-methylperlatolic acid (major) and perlatolic acid (minor or trace; reported only from *Chicitaealecanoriformis*). Spot tests: cortex K–, C–, KC–, P–, UV–; medulla and soralia K–, C–, KC–, P–, UV+ white.

**Figure 6.**
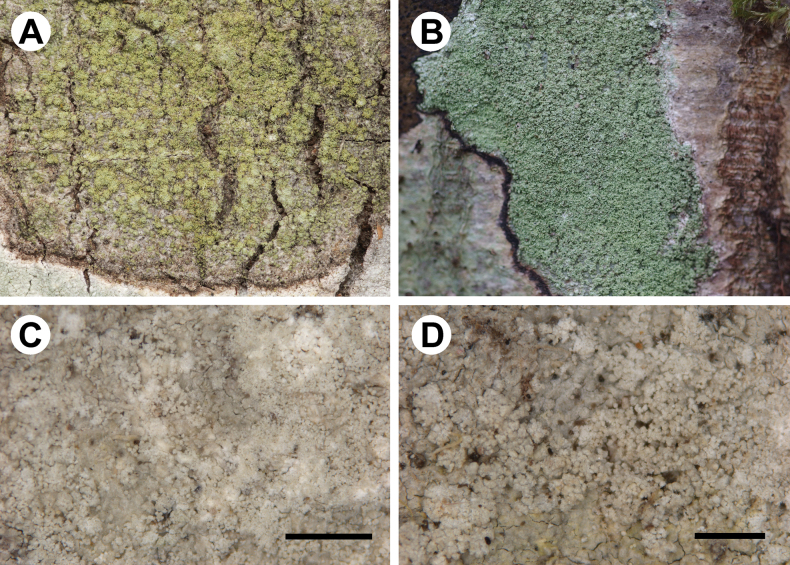
Morphology of two species of *Chicitaea***A** Thallus of *Ch.confusa* on tree trunk (taken by J. Hollinger in the field) **B** thallus of *Ch.cristinae* on tree trunk (taken by D. Kubiak in the field) **C, D** Thalli of *Ch.cristinae* showing soralia (paratypes of *L.cristinae***C** UGDA L-22396 **D** UGDA L-20385). Scale bars: 1 mm (**C, D**).

For morphology of *Chicitaea* species, see Lumbsch et al. (2007), Papong et al. (2009), Lendemer (2013), Guzow-Krzemińska et al. (2018) and Fig. 6.

#### ﻿ Chicitaea assateaguensis

Taxon classificationFungiSarrameanalesSarrameanaceae

(Lendemer) Guzow-Krzem., Kukwa & Lendemer comb. nov.

D05DE1B8-7444-5AEE-BE1B-93A2B54604CB

851780

Loxospora assateaguensis Lendemer, J. North Carolina Acad. Sci. 129(3): 74 (2013). Basionym.

#### ﻿ Chicitaea confusa

Taxon classificationFungiSarrameanalesSarrameanaceae

(Lendemer) Guzow-Krzem., Kukwa & Lendemer comb. nov.

2C4D221A-F24C-519F-B724-BF054C0BE3E1

851781

Loxospora confusa Lendemer, J. North Carolina Acad. Sci. 129(3): 77 (2013). Basionym.

#### ﻿ Chicitaea cristinae

Taxon classificationFungiSarrameanalesSarrameanaceae

(Guzow-Krzem., Łubek, Kubiak & Kukwa) Guzow-Krzem., Kukwa & Lendemer comb. nov.

4CF1C943-2AF0-59A9-BE6C-E3409177B57C

851782

Loxospora cristinae Guzow-Krzem., Łubek, Kubiak & Kukwa, in Guzow-Krzemińska, Łubek, Kubiak, Ossowska & Kukwa, Phytotaxa 348(3): 216 (2018). Basionym.

#### ﻿ Chicitaea lecanoriformis

Taxon classificationFungiSarrameanalesSarrameanaceae

(Lumbsch, A.W. Archer & Elix) Guzow-Krzem., Kukwa & Lendemer comb. nov.

C2C62C2C-5945-5AAF-856E-31E936E4A1CE

851783

Loxospora lecanoriformis Lumbsch, A.W. Archer & Elix, Lichenologist 39(6): 514 (2007). Basionym.

#### ﻿ Loxospora

Taxon classificationFungiSarrameanalesSarrameanaceae

A. Massal.

43353D09-7EF9-501B-AEBD-F1378F3D9546

Ric. Auton. Lich. Crost.: 137 (1852).

##### Notes.

Three species, *L.cyamidia* (Stirt.) Kantvilas, *L.septata* (Sipman & Aptroot) Kantvilas and *L.solenospora* (Müll. Arg.) Kantvilas (syn. *Sarrameanatasmanica* Vězda & Kantvilas), from the Southern Hemisphere have not been sequenced so far. However, they have ascospores similar in shape to other *Loxospora* spp. (although, in *L.cyamidia* and *L.solenospora*, they are rarely septate), asci with an amyloid apical dome and contain thamnolic acid (although *L.solenospora* may sometimes contain additionally gyrophoric acid or only the latter substance) (Kantvilas 2000, 2004). Given the morphological and chemical similarities to the type species *L.elatina* and other members of *Loxospora* s.str., they are treated here as belonging to this genus. *Loxosporaisidiata* Kalb (described from the Philippines) and *L.ochrophaeoides* Kalb & Hafellner (described from Madeira), introduced by Kalb and Hafellner (1992) and *L.glaucomiza* (Nyl.) Kalb & Staiger (described from Japan) treated by Staiger and Kalb (1995) are also treated as belonging to *Loxospora* s.str. due to the production of thamnolic acid.

The name *Loxosporapustulata* (Brodo & W.L. Culb.) Egan was applied to a common and widespread pustulose-sorediate crustose species with thamnolic acid that occurs throughout eastern North America (Brodo and Culberson 1986; Lendemer and Noell 2018). The discovery of fertile material led to its being transferred to the genus *Lepra* Scop. as *L.pustulata* (Brodo & W.L. Culb.) Lendemer & R.C. Harris (Lendemer and Harris 2017).

#### ﻿ Loxospora chloropolia

Taxon classificationFungiSarrameanalesSarrameanaceae

(Erichsen) Ptach-Styn, Guzow-Krzem., Tønsberg & Kukwa comb. nov.

1565BEC5-3C3B-51F0-A73C-950B0349D773

851745

Fig. 7

Pertusaria chloropolia Erichsen, in Zahlbr., Rabenh. Krypt.-Fl. Ed. 2, 9(5[1]): 645 (1935[1936]). Basionym. Type. [Switzerland. Jura Mts:] Mont de Baulmes, 1100 m elev., [on Abies] 1934, Meylan (lectotype: HBG!, selected here; MycoBank No: MBT 10017691).
Pertusaria chloropolia f. cana Erichsen, in Zahlbr., Rabenh. Krypt.-Fl. Ed. 2, 9(5[1]): 646 (1935[1936]). Syn. nov. Type. [Ukraine. Carpathians:] Lopušanka, 500 m elev., [corticolous] 1931, Nádvorník (lectotype: HBG!, selected here; MycoBank No: MBT 10017692).

##### Typifications.

The type specimen of *Pertusariachloropolia* consists of thin, continuous thallus with discrete soralia forming from flat parts of thalli or from slightly convex areoles and contains thamnolic acid (detected by I. M. Brodo). In the type specimen of P.chloropoliaf.cana, soralia are partly damaged, but, similarly to the type of *P.chloropolia*, the type consists of thin, continuous thallus with discrete soralia and contains thamnolic acid (detected by I. M. Brodo). In the protologue of P.chloropoliaf.cana, the type locality was cited as ‘Tschechoslowakei: Karpathoruβland, Lopusanka’ (Erichsen 1935), but to our knowledge, it is now located in western Ukraine. The name ‘Lopusanka’ is a spelling error as, on the label, it is ‘Lopušanka’.

**Figure 7.**
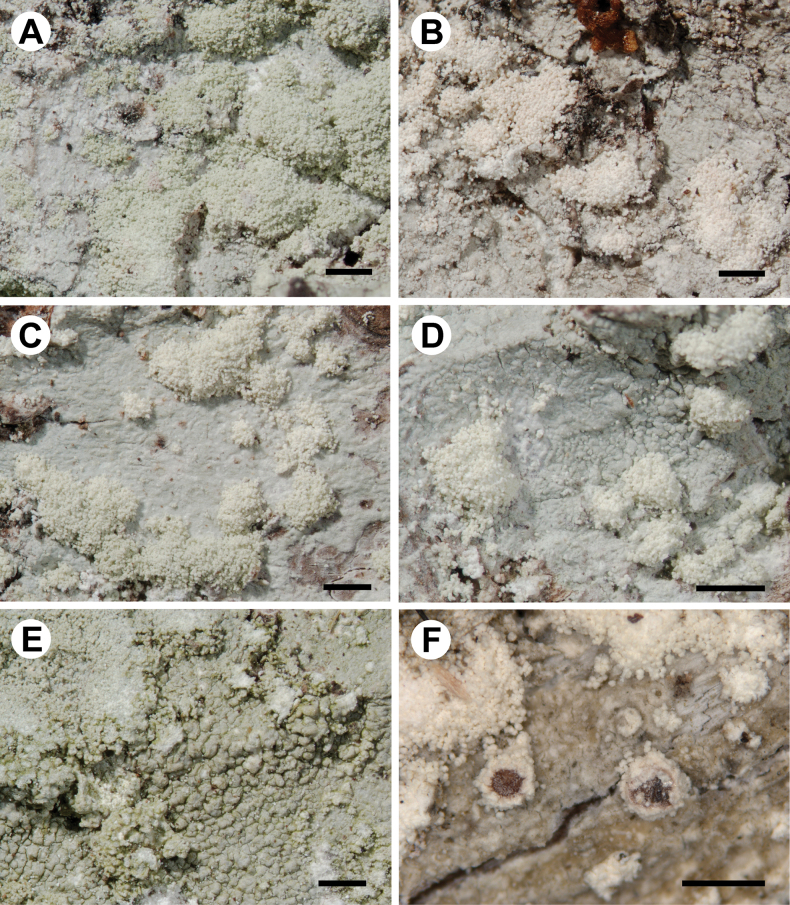
Morphology of *Loxosporachloropolia* (for details of specimens, see Table 1, Suppl. material 3) **A−C** smooth to folded thalli with mostly discrete soralia (**A** UGDA L-60095 **B** UGDA L-31983 **C** UGDA L-54253) **D, E** thalli with folded to areolate areas (**D** UGDA L-60093 **E** UGDA L-60096) **F** apothecia with sorediate margins (Ellis L456, E 01043201). Scale bars: 1 mm.

Erichsen (1935) cited only one locality for both names. However, the lectotypes are selected, because it is not known if, at the time of describing both taxa, C. F. E. Erichsen used only one element upon which the validating descriptions were based (Art. 9.3; Turland et al. (2018); see also McNeill (2014)).

##### Description.

Thallus crustose, grey, matt or more often shiny, thin, continuous, slightly folded, cracked to cracked areolate. Areoles flat or rarely convex, not constricted at the base. Soralia whitish to greenish-grey, flat or more often convex, rounded or irregular, mostly discrete and separated, bursting from flat parts of thallus or from areoles, sometimes crowded and the neighbouring soralia more or less fused, but still the boundaries often visible between them or, very rarely, soralia fused into irregular patches in older parts of thallus. Soredia up to 50 µm in diam., often in consoredia up to 100 µm wide. Apothecia very rare, single, up to 1.2 mm in diam. Thalline margin present, esorediate or partly to completely sorediate. Excipulum proporium not evident. Disc reddish-brown, thinly white pruinose. Hymenium up to 100 µm high. Epihymenium straw-brown (K+ pale reddish-brown), with dense granules dissolving in K. Paraphyses not capitate, sometimes anastomosing. Asci 8-spored, with uniformly KI+ blue apical dome. Ascospores 0–3(–5)-septate, spiralled in asci, hyaline, fusiform, curved, 35–48 × 5–7 µm. Pycnidia not known. Photobiont chlorococcoid, cells up to 12 µm in diam.

##### Chemistry.

Thamnolic acid (major), elatinic acid (minor, trace or absent) and squamatic acid (trace or absent). Spot tests: cortex, apothecial section, soralia and medulla K+ lemon-yellow, Pd+ yellow to orange, UV–.

##### Notes.

**Figure 8.**
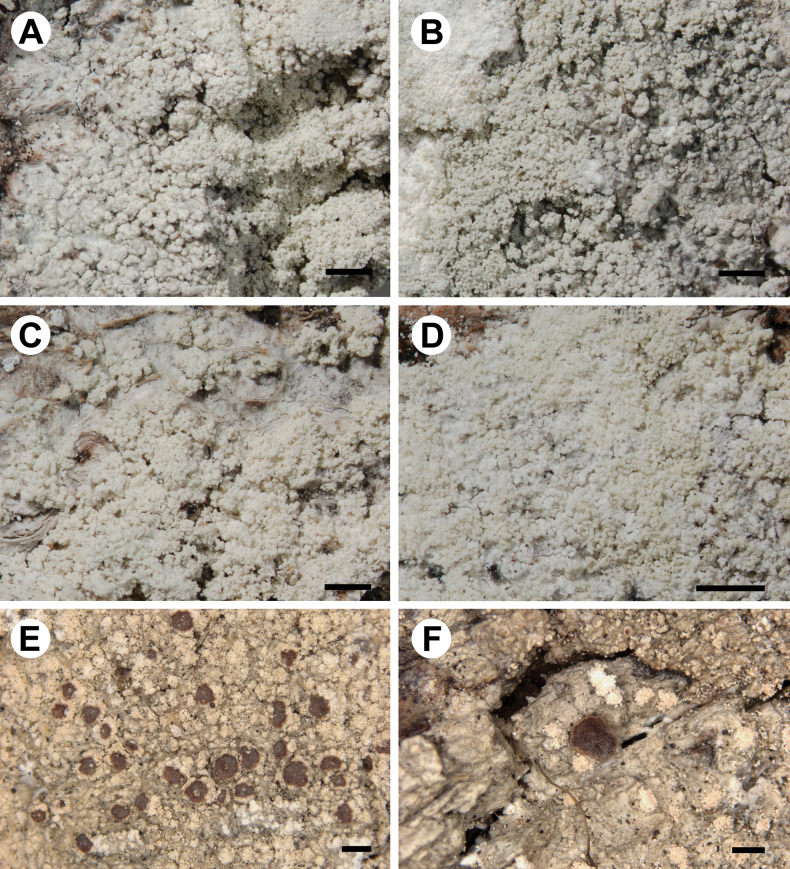
Morphology of *Loxosporaelatina* (for details of specimens, see Table 1, Suppl. material 3) **A, B** thalli with tuberculate areoles and irregular and partly fused soralia (**A** UGDA L-47757 **B** UGDA L- 47762) **C** thallus with soralia bursting from areoles and later fused (UGDA L-47761) **D** soralia covering most parts of the thallus (UGDA L-47760) **E, F** apothecia with sorediate or esorediate margins (O L-97759). Scale bars: 1 mm.

*Loxosporachloropolia* differs from *L.elatina* in having a thin, continuous to cracked-areolate thallus with mostly regular soralia, which are discrete at least in young parts of thalli (Fig. 7). Areoles in the central parts of larger thalli may become convex (in few specimens; Fig. 7E), but are never tuberculate or isidia-like as in *L.elatina* (Fig. 8). Soralia develop by breaking the cortex and are mostly regular, discrete and convex, rarely flat. Sometimes the neighbouring soralia are fused; however it is still possible to detect the boundaries between individual soralia in most cases. *Loxosporaelatina*, in contrast, has thalli which are, in most cases, tuberculate (sometimes only locally) or with areoles that resemble coarse isidia (Fig. 8). Tuberculate areoles are grouped or dispersed and constricted at the base. Soralia develop from the top of the tuberculate or pustulate areoles and are never regular as in *L.chloropolia* and, in most thalli, form granular-sorediate patches covering large areas (sometimes almost the entire thallus is covered with soredia; Fig. 8D). Moreover, these species differ in several nucleotide positions in both nuITS rDNA and RPB1 markers (Tables 2, 3).

*Loxosporachloropolia* can be confused with sorediate species of *Chicitaea*, but they contain 2’-*O*-methylperlatolic acid and the thallus is K negative (Lendemer 2013; Guzow-Krzemińska et al. 2018). *Lecanoranorvegica* Tønsberg is another similar species, which occurs on similar substrates, but it contains atranorin and protocetraric acid (Tønsberg 1992; Kukwa and Kubiak 2007).

##### Habitat and distribution.

The species is corticolous and grows in deciduous or mixed forests on bark of *Abiesalba*, *Acerpseudoplatanus*, *Alnusglutinosa*, *Betula* spp., *Corylusavellana*, *Fagussylvatica*, *Juniperuscommunis*, *Larixdecidua*, *Piceaabies*, *Pinussylvestris*, *Populustremula*, *Quercus* spp., *Sorbusaucuparia* and *Tiliacordata*. So far, it is known from Czechia, Great Britain, Latvia, Norway, Poland, Sweden, Switzerland (type locality) and Ukraine.

##### Specimens examined.

See Suppl. material 3.

#### ﻿ Loxospora elatina

Taxon classificationFungiSarrameanalesSarrameanaceae

(Ach.) A. Massal.

6578433A-AC9C-5555-B24F-2197538D6ECA

Fig. 8

Ric. Auton. Lich. Crost.: 138 (1852). – Lecanoraelatina Ach., Lich. Univ.: 387 (1810).

##### Type.

Lusatia, [corticolous], Mosig? (lectotype: H-ACH 1199A!, selected here; MycoBank No: MBT 10017693).

##### Typification.

In the protologue of *Lecanoraelatina*, Acharius (1810) cited the locality as “Habitat in cortice *Pini Abietis* Silesiae. Mosig”. The type collection in H-ACH consists of four pieces of bark covered with thalli of *Loxosporaelatina*. Three (H-ACH 1199A, 1199B and 1199C) are annotated “Lusatia” with a very faint pencil note next to H-ACH 1199A deciphered as possibly “Mosig” (this note probably not added by Acharius himself as the handwriting in pencil differs from all notes made in ink). The fourth specimen, H-ACH 1199D is annotated “Germania. Schrader”. According to the label added in modern times and attached to the type collection, Lusatia was part of Silesia, therefore, the three specimens annotated “Lusatia” can be considered original material; however, it is impossible to verify whether all three were collected by Mosig. Nevertheless, the largest sample (H-ACH 1199A) is fertile and apothecia were mentioned in the diagnosis, therefore it is selected as lectotype. The Acharius collection in BM also contains a specimen of *Lecanoraelatina*, however without any locality details; therefore, it cannot be considered as an isolectotype.

##### Description.

Thallus crustose, grey, matt, thin (at the margin) or more usually thick, continuous or cracked, slightly folded at least the margins, later areolate-verrucose to tuberculate (sometimes only part of the thallus tuberculate). Areoles usually strongly convex, tuberculate and constricted at the base or resembling coarse isidia, sometimes pustulate, dispersed or aggregated. Soralia whitish to greenish-grey, flat or more often convex, rounded or more often irregular, bursting from the top of areoles, often fused and tending to coalesce locally on the thallus or covering most parts of the thallus, sometimes developing from irregular cracks of the thallus. Soredia up to 60 µm in diam., often in consoredia up to 120 µm wide. Apothecia rare, up to 1.2 mm in diam., single or grouped up to five apothecia. Thalline margin present in young apothecia, smooth to flexuose, verrucose or dentate, sometimes with small soralia, later excluded. Excipulum proprium thin, flesh-coloured to white grey in surface view, orange-brown in section, smooth or more often flexuous, up to 100 µm wide in section. Disc reddish-brown, thinly white pruinose. Hymenium up to 125 µm high. Epihymenium straw-brown (K+ pale reddish-brown), with dense granules dissolving in K. Paraphyses not capitate, sometimes anastomosing. Asci 8-spored, with uniformly KI+ blue apical dome. Ascospores 0–5-septate, spiralled in asci, hyaline, fusiform, curved, 35–53(–64) × 4.5–6.5(–7) µm. Pycnidia not known. Photobiont chlorococcoid, cells up to 12 µm in diam.

##### Chemistry.

Thamnolic acid (major), elatinic acid (minor, trace or absent) and squamatic acid (trace or absent). Spot tests: cortex, apothecial section, soralia and medulla K+ lemon-yellow, Pd+ yellow to orange, UV–.

##### Notes.

*Loxosporaelatina* is similar to *L.chloropolia*; for differences, see under that species. The name (often as *Haematommaelatinum* (Ach.) A. Massal.) was often used in the past for the non-sorediate specimens currently referred to as *L.ochrophaea*. Both species, as mentioned above, are indeed morphologically (except for the production of soralia) and chemically almost identical and may represent the same species.

*Loxosporaochrophaeoides*, when described, was compared with *L.ochrophaea* and characterised as differing only in the presence of semi-globose soralia (Kalb and Hafellner 1992). Whether this taxon is distinct or synonymous with *L.elatina* or *L.chloropolia*, needs further studies using molecular techniques.

Some specimens of *L.elatina* were found to be determined as *Ochrolechiaandrogyna* (Hoffm.) Arnold, but that species and the recently segregated *O.bahusiensis* H. Magn. and *O.mahluensis* Räsänen differ in the production of gyrophoric acid and simple, larger ascospores (Tønsberg 1992; Kukwa 2011).

##### Habitat and distribution.

The species is corticolous or lignicolous and grows on bark of various coniferous and deciduous tree in forests. The species was reported from many countries in the Northern Hemisphere; however, as some records may belong to *L.chloropolia*, its distribution needs revision. In the course of this study, we examined specimens from Austria, Czechia, Estonia, Finland, Latvia, Lithuania, Poland, Slovakia, United Kingdom, Ukraine and USA.

##### Specimens of *Loxosporaelatina* and *L.ochrophaea* examined.

See Suppl. material 3.

## Supplementary Material

XML Treatment for Chicitaea

XML Treatment for Chicitaea assateaguensis

XML Treatment for Chicitaea confusa

XML Treatment for Chicitaea cristinae

XML Treatment for Chicitaea lecanoriformis

XML Treatment for Loxospora

XML Treatment for Loxospora chloropolia

XML Treatment for Loxospora elatina

## ﻿Additional information

### Author ORCIDs

Łucja Ptach-Styn https://orcid.org/0000-0003-2027-1636

Beata Guzow-Krzemińska https://orcid.org/0000-0003-0805-7987

James C. Lendemer https://orcid.org/0000-0003-1186-0711

Martin Kukwa https://orcid.org/0000-0003-1560-909X

### Data availability

All of the data that support the findings of this study are available in the main text or Supplementary Information.

## Supplementary materials

Supplementary material 1 Conditions for each set of primers used in PCR Data type docx https://binary.pensoft.net/file/986579This dataset is made available under the Open Database License (http://opendatacommons.org/licenses/odbl/1.0/). The Open Database License (ODbL) is a license agreement intended to allow users to freely share, modify, and use this Dataset while maintaining this same freedom for others, provided that the original source and author(s) are credited.Łucja Ptach-Styn, Beata Guzow-Krzemińska, James C. Lendemer, Tor Tønsberg, Martin Kukwa

Supplementary material 2 Sequences obtained from GenBank and used in phylogenetic analyses Data type xlsx Explanation note Samples marked with an asterisk in herbarium column were revised by authors. https://binary.pensoft.net/file/986580This dataset is made available under the Open Database License (http://opendatacommons.org/licenses/odbl/1.0/). The Open Database License (ODbL) is a license agreement intended to allow users to freely share, modify, and use this Dataset while maintaining this same freedom for others, provided that the original source and author(s) are credited.Łucja Ptach-Styn, Beata Guzow-Krzemińska, James C. Lendemer, Tor Tønsberg, Martin Kukwa

Supplementary material 3 Specimens of *Loxosporachloropolia*, *L.elatina* and *L.ochrophaea* revised for this study Data type xlsx https://binary.pensoft.net/file/986581This dataset is made available under the Open Database License (http://opendatacommons.org/licenses/odbl/1.0/). The Open Database License (ODbL) is a license agreement intended to allow users to freely share, modify, and use this Dataset while maintaining this same freedom for others, provided that the original source and author(s) are credited.Łucja Ptach-Styn, Beata Guzow-Krzemińska, James C. Lendemer, Tor Tønsberg, Martin Kukwa
