# Supplementary material for: ﻿Phylogeny of the genus Loxospora s.l. (Sarrameanales, Lecanoromycetes, Ascomycota), with Chicitaea gen. nov. and five new combinations in Chicitaea and Loxospora
Source: MycoKeys. 2024 Feb 19;102:155–81. doi: 10.3897/mycokeys.102.116196 (PMC10897838; doi:10.3897/mycokeys.102.116196)
Supplement: Supplementary material 1 — Conditions for each set of primers used in PCR [file mycokeys-102-155-s001.docx]

Conditions for each set of primers used in PCR.

| Set of primers | Details of program |
| --- | --- |
| ITS1F and ITS4; ITS5 and ITS4 | 1. initial denaturation: 95°C, 3min 2. 35 cycles:  - denaturation: 95°C, 30s - annealing: 54°C, 30s - extension: 72°C, 1min  1. final extension: 72°C, 10min |
| mrSSU1 and mrSSU3R | 1. initial denaturation: 95°C, 3min 2. 6 cycles:  - denaturation: 95°C, 1min - annealing: 62°C, 1min - extension: 72°C, 1min 30s  1. 33 cycles:  - denaturation: 94°C, 1min - annealing: 56°C, 1min - extension: 72°C, 1min  1. final extension: 72°C, 7min |
| Lox_mtSSU620_For and Lox_mtSSU620_Rev | 1. initial denaturation: 95°C, 3min 2. 35 cycles:  - denaturation: 95°C, 30s - annealing: 51°C, 45s - extension: 72°C, 30s  1. final extension: 72°C, 10min |
| g-RPB1-A for and f-RPB1-C rev | 1. initial denaturation: 92°C, 2min 2. 7 cycles:  - denaturation: 94°C, 1min - annealing: 60°C, 1min 30s - extension: 72°C, 1min 45s  1. 33 cycles:  - denaturation: 94°C, 1min - annealing: 56°C, 1min 30s - extension: 72°C, 2min  1. final extension: 72°C, 10min |
